# Do Psoriasis and Atopic Dermatitis Affect Memory, Attention, Stress and Emotions?

**DOI:** 10.3390/brainsci14080747

**Published:** 2024-07-25

**Authors:** Marcin Kuryłło, Ewa Mojs, Natalia Woźniak, Dorota Wiśniewska-Szeplewicz

**Affiliations:** Department of Clinical Psychology, Poznan University of Medical Sciences, 60-812 Poznań, Poland; marcin@kuryllo.pl (M.K.);

**Keywords:** cognitive functions, memory, attention, emotions, psoriasis, atopic dermatitis

## Abstract

Background: Psoriasis and atopic dermatitis are chronic skin diseases found all over the world that cause a lot of suffering to patients. Objectives: The aim of this study was to answer the following questions: whether people suffering from psoriasis and AD have greater problems with recognizing emotions, the effectiveness of attention and memory processes, and whether they use different strategies of coping with stress than healthy people. Methods: This study involved 90 patients, including 30 patients with psoriasis, 30 patients with AD and 30 healthy patients, aged 21 to 63 years, including 54 women and 36 men. This study used a battery of the CANTAB Cognitive Tests, Mini-COPE Questionnaire Inventory, Toronto Alexithymia Scale TAS Questionnaire, Psoriasis Area and Severity Index, and Eczema Area and Severity Index. Results: People with psoriasis and AD had higher total scores on the alexithymia scale and had greater difficulty in identifying and verbalizing emotions. People with psoriasis and AD are less likely to choose the correct stimulus and achieve a shorter length of the sequence that should be remembered. Psoriasis patients with more severe symptoms are less likely to use the strategy of a sense of humor in stressful situations. AD patients with more severe symptoms are less likely to use strategies of operative thinking, denial and self-blame, and the strategy of seeking instrumental support is used more often. Conclusions: Patients with psoriasis and AD require a holistic approach; in addition to dermatological treatment, psychological support, psychotherapeutic support and possible psychiatric treatment are recommended.

## 1. Introduction

Psoriasis and atopic dermatitis are frequent chronic skin diseases present globally, with the morbidity growing year to year [1]. Psoriasis is a chronic, autoimmune, non-inflammatory skin disease with unpredictable course of symptoms, a range of external factors and comorbidities, including but not limited to joints inflammation, circulatory system diseases, metabolic syndrome, inflammatory bowel disease, and mental health disorders [2,3]. According to World Health Organization data, psoriasis concerns people of all ages and all over the world. The prevalence of psoriasis in respective countries ranges from 0.09% to 11.43% which means that psoriasis is a major global problem affecting at least 100 million people all over the world [2]. In Poland, over 1 million people suffer from psoriasis [4].

Atopic dermatitis is a chronic and recurrent inflammatory skin disease of complex etiology [5] which is a significant burden for healthcare resources [6]. It is estimated that the direct cost of atopic dermatitis in the United States alone amounts to nearly 1 billion US dollars a year [7]. According to data supplied by the WHO Global Burden of Diseases initiative, it is estimated that AD affects at least 230 million people globally [8,9,10]. The disease affects about 15–30% of children and 2–10% of adults [11,12]. Increasingly, apart from the medical aspects of the disease, there is a growing interest in how the disease affects the quality of life of patients [13,14,15,16,17]. It is noted that AD patients are more susceptible to the risk of infections, cardiovascular diseases [18] and many neuropsychiatric disorders, including depression and Attention Deficit Hyperactivity Disorder (ADHD), speech disorders in childhood, headaches and convulsions. There is also a multi-factor association between AD and osteoporosis, traumas and fractures [19].

Over the recent years, there have been many studies on the existence of a relationship between the occurrence of psoriasis and AD, and mental condition of patients [20,21,22,23,24]. According to a systematic review by Ferreira et al. (2016), psoriasis is linked with many mental disorders, both in the psychotic and neurotic spectrum. Chronic stress diminishes hypothalamic–pituitary–adrenal axis and upregulates sympathetic–adrenal–medullary responses, stimulating pro-inflammatory cytokines. Then, it maintains and exacerbates psoriasis and some of its mental disorders [25]. Chida et al. (2007) conducted a systematic review and meta-analysis of 43 studies that assessed the influence of psychosocial factors on atopic disorders and the influence of atopic disorders on mental health. The review revealed a strong association between psychosocial factors and atopic disorders [26]. Cao S. et al. (2024) emphasized the significant causal association between AD and an increased risk of autism spectrum disorder and also identified bipolar disorder and anorexia nervosa as risk factors for atopic dermatitis [27]. Scientific reports in this area are ambiguous. In an observational pilot study by Emal et al. (2024), the authors did not find significant differences in the subjective estimate of life quality in the domains of physical health, psychological health, social relations, or environment between non-atopic subjects; atopics without allergic symptoms; and atopics with allergic symptoms. These results may indicate that asymptomatic atopy, as well as relatively mild allergy symptoms, does not affect the quality of life at all [28]. Radosević-Vidacek et al. (2009), in their study on a sample of active workers, also found no association of neuroticism, exposure to stressful life events, and quality of life with atopy and allergic symptoms [29]. The prevalence of mental problems among dermatological patients is estimated at 30–60% [30]. The most frequent mental disorders in patients with psoriasis and AD are depression and anxiety disorders. Psoriasis is associated not only with anxiety disorders, depression and suicidal thoughts but also with considerable impairment of cognitive processes, including attention, visual and verbal memory and executive functions [31,32,33,34,35]. In the case of atopic dermatitis, some researchers highlight the links between the prevalence of this dermatosis and impairment of cognitive processes, including attention, visual and verbal memory, executive functions, and abstract thinking [36,37]. Patients with atopic dermatitis are also more prone to experience alexithymia disorders [36].

Due to such a large scale of prevalence of both diseases and the negative effects they exhibit in the field of mental disorders, as well as the ambiguous results of previous studies in this area, it seems justified to conduct further research assessing how psoriasis and atopic dermatitis affect human functioning in everyday life, including the strategies used to cope with stress, experiencing emotions and cognitive processes. 

The aim of this study was to answer the question whether people suffering from psoriasis and atopic dermatitis have greater problems with recognizing emotions, the effectiveness of attention and memory processes, and whether they use different strategies of coping with stress than healthy people. The authors would also like to know whether being overweight, in all study groups, is associated with cognitive processing, emotions, and stress coping. The answer to this question may contribute to a better understanding of the problems faced by people chronically ill with skin diseases and consequently improve the quality of medical care by adding appropriate psychological and psychiatric interventions to dermatological treatment.

## 2. Materials and Methods

### 2.1. Participants and Procedure

This study involved 90 patients, including 30 patients with psoriasis, 30 with atopic dermatitis and 30 healthy patients, aged 21 to 63 years (*M* = 37.00; *SD* = 10.51), including 54 women (60%) and 36 men (40%). Healthy subjects constituted the control group. The inclusion criteria for this study included age between 18 and 65 years, diagnosis of psoriasis or atopic dermatitis confirmed by a dermatologist, and informed consent to participate in this study. Exclusion criteria were age below 18 years or over 65 years, no psoriasis or atopic dermatitis, or central nervous system disease. Psychodemographic data of the study groups and the control group are presented in Table 1. Before taking the tests, the participants completed a study protocol in which they provided the following information: medical diagnosis of skin diseases, age, education, professional status, place of residence, body weight, height and comorbidities. The above variables were taken into account in the analyses. Body mass and height declared by the subjects were used for the calculation of body mass index (BMI) in compliance with the guidelines of the World Health Organization (WHO). All the subjects gave informed consent to participate in this study. This study was approved by the Bioethics Committee at Poznan University of Medical Sciences (Resolution No. 24/21 of 14 January 2021).

### 2.2. Research Tools

The following tools were used in this study:

The PASI (Psoriasis Area and Severity Index) questionnaire used to assess the severity of psoriasis. It is the index taking into account the extent and severity of skin lesions. It determines erythema, induration and desquamation on a scale from 0 (no lesions) to 4 (very severe lesions) and the affected area in four locations: the head, trunk, upper limbs, and lower limbs in the range from 0 (<10%) to 6 (90–100%). The maximum score on the scale is 72. The higher the score, the greater the severity of psoriasis [38]. Despite the fact that the PASI also has drawbacks, it is the most appropriate tool available for assessing the severity of plaque psoriasis [39]. 

EASI questionnaire (Eczema Area and Severity Index) created by Hanifin et al. [40]. It takes into account the extent and severity of skin lesions and ignores subjective symptoms such as itching or sleep disorders. It defines each of the four symptoms: erythema, infiltration, excoriations and lichenization, on a scale from 0 (no lesions) to 3 (very severe lesions) in four locations—the head, trunk, upper limbs, and lower limbs—and the affected area in these areas are in a range from 0 (no lesions) to 6 (90–100%). The maximum score on the scale is 72 [5]. In line with Leshem et al., the following interpretation of the EASI result was adopted: 0 = clear, 0.1–1.0 = almost clear, 1.1–7.0 = mild, 7.1–21.0 = moderate, 21.1–50.0 = severe, and 50.1–72.0 = very severe [5,41]. 

The Mini-COPE Questionnaire Inventory for assessing coping with stress was authored by Charles S. Carver, and the authors of the Polish adaptation are Zygfryd Juczyński and Nina Ogińska-Bulik. The questionnaire is used to assess methods of coping with stress. It consists of 28 statements that are part of 14 strategies (2 statements in each strategy): active coping, planning, positive reinterpretation, acceptance, sense of humor, turning to religion, seeking of emotional support, seeking of instrumental support, doing something else, denial, venting of emotions, use of psychoactive substances, cessation of activities, and self-blame [42].

The Toronto Alexithymia Scale TAS Questionnaire—20 PL was authored by Taylor G.J., Bagby R.M., and Parker J.D.A. (1994), and the authors of the Polish adaptation are Ścigała D.K., Zdankiewicz-Ścigała E., and Bedyńska S. Kokoszka A. (2020). Mattila et al. found that the rates of alexithymia in the general population are 9–17% for men and 5–10% for women [43,44]. The questionnaire is used to measure alexithymia, understood as a difficulty in recognizing, processing and regulating emotions. It consists of 20 statements that are part of 3 sub-scales: difficulty identifying emotions (DIF), difficulty describing emotions (DDF), and operational thinking style (EOT). The TAS Total is the sum of the respective strategies, i.e., TAS Total = DIF + DDF + EOT [45]. A score below 51 points indicates no alexithymia, a score equal to or greater than 61 indicates alexithymia, and a score between 52 and 60 points indicates the possible presence of alexithymia [46].

A battery of CANTAB Cognitive Tests intended for the assessment of cognitive processes, developed by Cambridge Cognition (CANTAB^®^ Cognitive assessment software. Cambridge Cognition 2023. All rights reserved; https://cambridgecognition.com/digital-cognitive-assessments/ (accessed on 1 December 2023)). 

The following tools were used for testing attention and psychomotoric speed:

Rapid Visual Information Processing (RVP)—A test for rapid visual information processing is a measure of attention maintenance. It takes 7 min to complete the test. Participants are asked to detect target sequences of three digits. When a participant sees the target sequence, they must respond by selecting the button in the center of the screen as quickly as possible. 

The following methods were used to study memory: 

Paired Associates Learning (PAL)—This test assesses visual memory and learning. It takes 8 min to complete the test. Fields with images are displayed around the screen in random order. The patterns are then displayed in the center of the screen, one by one, and the participant must select the field where the pattern was originally located.

Pattern Recognition Memory (PRM)—This test assesses patterns recognition memory. It takes 4 min to complete the test. A participant is given a series of visual patterns, one by one, in the center of the screen. In the recognition phase, the participant must choose between a pattern they have already seen and a new pattern. The second phase of recognition is administered after a 30 min delay.

Spatial Span (SSP)—This test evaluates the capacity of spatial working memory. It takes 5 min to complete the test. White squares are displayed on the screen, some of which briefly change color in a variable order. The participant must then check the boxes that have changed color in the same order in which they were displayed by the computer. The number of squares in the sequence increases from two at the beginning of the test to nine at the end, and the order and color change over the course of the test.

To study the functions of thinking and problem solving, the following was used:

One Touch Stocking (OTS)—This test assesses planning and spatial working memory. It takes 10 min to complete the test. OTS is based upon the Tower of Hanoi test. The participant must find out how many moves are required to find the solution.

To recognize emotions, the following was used:

Emotion Recognition Task (ERT)—This test assesses the ability to identify six basic emotions in face mimics. It takes 6 min to complete the test. Faces are displayed on the screen; each face is displayed for 200 ms and then is immediately covered. The participant must choose which emotion was displayed on the face from six options (sadness, happiness, fear, anger, disgust, or surprise).

A description of the variables is given in Table 2. IBM SPSS Statistic 28 was used to perform the statistical analysis. The level of statistical significance was assumed to be *p* < 0.05.

## 3. Results

Due to the vast majority of skewed distributions of variables, nonparametric tests were used for calculations: the Kruskal–Wallis test and Spearman correlation test.

PASI vs. TAS-20, Mini-COPE and CANTAB:

The results of the Spearman correlation test indicated the existence of a significant negative, moderate association between the PASI and sense of humor; the higher the psoriasis severity index, the less frequently the humor strategy was used.

EASI vs. TAS-20, Mini-COPE and CANTAB:

The results of the Spearman correlation test indicated the existence of significant negative, moderate associations between AD severity (EASI) and TAS-20 TOTAL, EOT, denial, self-blame and RVPA. 

The greater the severity of AD symptoms,

- the lower the total score on the alexithymia scale;
- the less frequently used the strategy of the operational mindset;
- the strategy of denial and self-blame;
- the less correctness was demonstrated in detecting the target pattern.

The results of the test also indicate the existence of significant positive, moderate associations between the EASI and the seeking of instrumental support and PRMPCI. 

The greater the severity of AD,

- the more often the strategy of seeking specific help was used;
- the more often the subjects indicated the correct patterns in the situation of immediate choice.

Health condition vs. TAS-20, Mini-COPE and CANTAB:

The results of the Kruskal–Wallis H test presented in Table 3 indicate the occurrence of significant differences due to the health condition in terms of total score on the alexithymia scale (TAS20 TOTAL), difficulty identifying feelings (DIF), difficulty describing feelings (DDF), delay in choosing emotions (ERTRT), number of correct patterns selected by the examined person in the state of delayed (PRMPCD) and immediate (PRMPCI) stimulus selection, correctness of the detection of the target stimulus (RVPA) and the longest sequence of fields successfully recalled by the subject (SSPFSL). 

People with psoriasis and atopic dermatitis had

- higher total scores on the alexithymia scale;
- greater difficulty in identifying emotions;
- greater difficulty verbalizing emotions;
- more accurate target stimulus detection

than healthy subjects.

People with psoriasis have greater delays in choosing emotions than people with AD and healthy people. 

People with psoriasis and atopic dermatitis

- are less likely to choose the correct stimulus;
- achieve a shorter length of the sequence that should be remembered

than healthy people.

BMI PASI vs. TAS-20, Mini-COPE and CANTAB:

The results of the Spearman correlation test presented in Table 4 indicate the existence of significant positive, moderate relationships between the BMI of people with AD and the total number of points on the alexithymia scale (TAS20 TOTAL), difficulties in verbalizing emotions (DDF), difficulties with the operational thinking style (EOT) and the strategy of taking psychoactive substances.

The higher the BMI in people with AD,

- the higher the total number of points on the alexithymia scale;
- the greater the difficulties in verbalizing emotions;
- the greater the difficulties with the operational thinking style;
- the more frequent taking of psychoactive substances.

The results of the test indicate the existence of significant positive, moderate relationships between the BMI in healthy people and the strategy of sense of humor. The higher the BMI in healthy people, the more often they use the strategy of sense of humor.

The results of the test also indicate significant negative, moderate relationships between the BMI in healthy people and the strategies of seeking emotional support, taking psychoactive substances and immediate selection situation (PRMPCI).

The higher the BMI in healthy individuals,

- the less frequent the search for emotional support;
- the less frequent the use of psychoactive substances;
- the slower the selection of a stimulus.

Alexithymia and cognitive processes:

Among people with psoriasis:

- the higher the score on the alexithymia scale, the fewer correct patterns selected in the situation of immediate selection situation (PRMPCI);
- greater difficulty in verbalizing emotions is associated with fewer correctly selected patterns in an immediate selection of the stimulus;
- the more often the operational thinking style is used, the lower the delay in choosing emotions after receiving the stimulus (ERTRT) and the fewer correctly selected patterns in the immediate selection situation.

Among people with AD:

- the higher the score on the alexithymia scale, the fewer cases in which the subject chose the correct field during the first attempt (PALFAMS);
- the higher the average number of attempts needed to successfully complete the stage (PALTEA), the fewer correctly selected patterns in the immediate selection situation (PRMPCI);
- greater difficulty in identifying emotions is associated with fewer correctly selected patterns in an immediate selection situation (PRMPCI) and a shorter sequence of successfully recalled fields (SSPFSL);
- greater difficulty in verbalizing emotions is associated with a greater average number of attempts needed to successfully complete the stage (PALTEA);
- the more often the operational thinking style is used, the lower the total number of correctly selected emotions (ERTTH);
- the fewer times the subject chose the correct field on the first attempt (PALFAMS), the higher the average number of attempts needed to successfully complete the stage (PALTEA);
- the lower the number of correctly selected patterns in the immediate selection situation (PRMPCI).

Among healthy subjects, there were no correlations between the alexithymia score and cognitive functions that would be statistically significant.

## 4. Discussion

Memory, attention and emotions can be affected by many factors, among other everyday events: the degree of stimulation of the body, stress, fatigue, medications taken, and hormonal disorders. For this reason, it is difficult to accurately measure the influence of chronic skin diseases, including psoriasis and atopic dermatitis on cognitive processes and emotions. Most of the studies to date have been devoted to the impact of psoriasis and atopic dermatitis on the quality of life of patients and mental disorders, with particular emphasis on depression and anxiety disorders. Although there are new scientific reports [47,48,49,50,51], there is still a lack of extensive research on the impact of psoriasis and AD on cognitive processes. The team of Marek et al. assessed the cognitive functions of patients with psoriasis, related to the function of the prefrontal cortex of the brain. Researchers have shown that patients with psoriasis perform worse than healthy people on neuropsychological tests assessing working memory and executive functions [31]. In a study conducted on 200 people, Padma et al. showed that patients with psoriasis had cognitive deficits in attention, concentration, and total scores on the Standard Mini-Mental Status Examination (SMMSE) and the Brief Cognitive Rating Scale (BCRS) tests used to assess cognitive functions [32]. In the case of atopic dermatitis, the links between the occurrence of this dermatosis and impairment of cognitive processes, including attention, visual and verbal memory, executive functions and abstract thinking were identified [36].

The authors’ own study using the CANTAB Cognitive Tests showed that people with psoriasis and atopic dermatitis have a worse memory of recognizing patterns and remember a shorter length of the sequence of stimuli presented consecutively (spatial working memory) than healthy people. These results are consistent with previous studies. What is less consistent seems to be the results in which subjects with psoriasis and atopic dermatitis showed better performance in terms of maintained attention in the test for rapid processing of visual information (strings of digits). 

Researchers have shown that AD patients with more severe symptoms of the disease processed visual information more slowly but showed better visual memory when stimuli were immediately reproduced.

There are studies indicating that alexithymia may be the result of long-term stress associated with psoriasis and depression [52]. Some authors prove much higher prevalence and severity of alexithymia in patients with psoriasis and atopic dermatitis [1,24,36]. The link between alexithymia and various medical disorders, including psoriasis, suggests that it may be a risk factor for their development. In particular, people with alexithymia do not seem to cope effectively with stressors due to stress response that is usually altered in cognitive processing (lack of emotional awareness) [53]. According to Baysak et al. (2020), the correlation between alexithymia and passive coping styles with ruminations may affect the course of psoriasis [54]. In the present study, the authors showed that people with psoriasis and atopic dermatitis have greater difficulty identifying and verbalizing emotions. People with psoriasis recognize emotions with greater delay than people with AD and healthy people. AD patients with more severe symptoms of the disease have a lower rate of alexithymia and are less likely to use operative mindset strategies. The authors also compared the severity of alexithymia to the results of cognitive tests. Comparisons between the groups showed differences. Correlations between alexithymia and cognitive processes occurred both among patients with psoriasis and AD, while no correlations were found in healthy subjects. It appears that chronic skin disease can negatively affect the ability to respond emotionally in people with psoriasis and atopic dermatitis, and this in turn can cognitively distort reality. It is not known what the direction of the relationship is and whether it is unidirectional or bidirectional, so further research in this area is required. 

It should be noted that the severity of symptoms among people with psoriasis and AD was small. For psoriasis, the lowest PASI score was 0.20, and the highest was 10.10 (mean 2.05; SD 2.16). Whereas for AD, the lowest EASI score was 0.20 and the highest was 15.40 (mean 2.30; SD 3.26). When analyzing the results, it is worth remembering that the EASI scale equally takes into account the extent and severity of the symptoms of the disease, so there may be a heterogeneous population of patients with the same EASI score [55,56]. Also, the PASI scale has its limitations; it is difficult to interpret because the relationship between the PASI score and the severity of the disease is non-linear. It is also not very sensitive to changes in the case of small skin coverage, which may distort the assessment of the effectiveness of the treatment [38]. The assessment of disease eruptions was subjectively reported by the patients; the researchers did not observe individual parts of the patients’ bodies.

Many studies to date have shown that obesity is strongly associated with the onset and exacerbation of psoriasis [57,58,59,60]. In patients with psoriasis, the prevalence of obesity is much higher [61,62], and the risk of obesity is higher [63,64,65]. The results of studies on the relationship between obesity and atopic dermatitis are heterogeneous [66]. The authors of this study did not show a significant correlation between BMI and psoriasis and atopic dermatitis, but they showed that overweight among people with AD is associated with greater difficulties in visualizing emotions, is associated with greater difficulties with the operative style of thinking and is more often associated with psychoactive substance abuse.

Many researchers point out that smoking and alcohol consumption are linked to psoriasis [57]. A systematic review and meta-analysis have shown that patients with psoriasis are more likely to smoke or have smoked in the past [67]. Smoking increases the risk of developing psoriasis [68]. Alcohol consumption appears to be a risk factor for psoriasis. Higher alcohol consumption was observed in patients with psoriasis than in the general population. Alcohol abuse is positively correlated with the severity of psoriasis and reduced treatment effectiveness [57,69,70].

The authors of this study also attempted to investigate whether smoking and alcohol consumption were associated with psoriasis and AD, but the groups were too heterogeneous to make a reliable assessment.

The main limitations of this study are its small sample size (n = 90); difficulty having access to the study participants (patients with psoriasis and atopic dermatitis often feel ashamed and are reluctant to share information about their health); and heterogeneity of the groups in terms of variables such as comorbidities, smoking, and alcohol consumption.

## 5. Conclusions

1. Individuals with psoriasis and AD have worse pattern recognition memory and remember shorter sequences of stimuli compared to healthy people.
2. Chronic skin diseases like psoriasis and AD can negatively impact emotional responsiveness.
3. Patients with severe atopic dermatitis (AD) have lower levels of alexithymia, are less likely to use operative thinking, denial, and self-blame strategies, and process visual information more slowly. They also more frequently seek instrumental support and show better immediate visual memory for stimuli.
4. People with psoriasis and AD have greater difficulty identifying and verbalizing emotions but maintain attention better than healthy individuals. Psoriasis patients recognize emotions more slowly than those with AD and healthy individuals.
5. Patients with severe psoriasis are less likely to use humor to cope with stress.
6. Overweight individuals with AD have more difficulty visualizing emotions, struggle more with operative thinking and are more likely to abuse psychoactive substances.
7. Overweight healthy individuals have a greater sense of humor, use psychoactive substances less frequently and rely less on emotional support from close relationships. As body weight increases, visual memory declines.
8. Patients with psoriasis and AD need a holistic approach, including dermatological treatment, modern biological therapies, psychological and psychotherapeutic support, and psychiatric care.

## Figures and Tables

**Table 1:** Psychodemographic data by group.

Variable		Patients with Psoriasis (n = 30)	Patients with AD (n = 30)	Healthy Subjects (n = 30)
age	average	40.20	37.53	33.27
	SD	11.15	9.51	9.94
sex	females	16	18	20
	males	14	12	10
BMI	average	29.28	24.76	23.17
	SD	6.61	4.38	3.24
education	primary school	-	2	-
	vocational school	1	1	-
	secondary school	9	11	2
	university	20	16	28
professional status	employed	30	26	30
	unemployed	-	4	-
place of residence	city < 50 thousand inhabitants	18	12	1
	city 50–200 thousand inhabitants	7	8	-
	city > 200 thousand inhabitants	5	10	29

**Table 2:** Description of the variables in the CANTAB Cognitive Tests battery.

Variable	Description
ERTRT	Median delay in choosing an emotion after receiving the stimulus
ERTTH	Total number of correctly chosen emotions
OTSMDLFC	Median delay measured from the appearance of the balls to the moment the subject makes the first choice of solution
PALFAMS	Number of times the subject selected the correct field on the first attempt
PALMETS	Average number of attempts needed to successfully complete a stage
PALTEA	The number of times the subject selected the wrong field, plus the estimated number of errors they would have made for problems they did not reach
PRMPCD	Number of correct patterns selected by the subject in the delayed selection state
PRMPCI	Number of correct patterns selected by the subject in the immediate selection state
RVPA	A measure of the correctness of the detection of the target stimulus
SSPFSL	Longest sequence of fields successfully recalled in the correct order

Source: own study.

**Table 3:** Health condition vs. TAS, Mini-COPE and CANTAB: Kruskal–Wallis H test and post hoc Dunn test.

Variable	Health Condition						Post Hoc	*H*	*p*
	Psoriasis		AD		Healthy Subjects				
	*M*	*SD*	*M*	*SD*	*M*	*SD*			
**TAS20 TOTAL**	**52.30**	**12.75**	52.86	14.48	44.50	8.55	1.2 > 3	6.91 *	0.032
**DIF**	18.83	6.37	20.21	7.46	15.37	5.14	1.2 > 3	7.27 *	0.026
**DDF**	14.23	4.39	14.34	5.53	11.37	4.41	1.2 > 3	7.16 *	0.028
**EOT**	19.23	4.66	18.31	5.32	17.77	3.67	-	1.75	0.417
**Active coping**	2.07	0.68	2.30	0.61	2.23	0.58	-	1.48	0.476
**Planning**	2.15	0.49	2.38	0.52	2.32	0.58	-	3.11	0.211
**Positive reinterpretation**	1.63	0.72	1.88	0.84	1.77	0.85	-	1.59	0.452
**Acceptance**	1.83	0.75	2.02	0.68	1.90	0.74	-	0.76	0.684
**Sense of humor**	0.92	0.59	1.03	0.73	0.90	0.62	-	0.33	0.848
**Turning to religion**	0.40	0.66	0.70	0.98	0.42	0.67	-	2.39	0.303
**Seeking of emotional support**	1.72	0.88	1.90	0.99	1.80	0.74	-	0.42	0.809
**Seeking of instrumental support**	1.67	0.81	1.78	0.87	1.80	0.65	-	0.36	0.834
**Doing something else**	1.60	0.67	1.93	0.75	1.77	0.69	-	3.54	0.170
**Denial**	0.62	0.58	0.82	0.89	0.48	0.52	-	1.61	0.448
**Venting of emotions**	1.28	0.69	1.55	0.75	1.47	0.63	-	2.08	0.353
**Use of psychoactive substances**	0.38	0.54	0.38	0.63	0.23	0.47	-	1.73	0.421
**Cessation of activities**	0.68	0.66	0.90	0.80	0.62	0.60	-	1.62	0.445
**Self-blame**	1.45	0.80	1.50	0.88	1.50	0.87	-	0.02	0.988
**ERTRT**	2111.17	1338.28	1756.77	974.45	1449.23	368.35	1 > 2.3	5.94 *	0.050
**ERTTH**	31.40	3.95	30.77	3.04	29.97	3.23	-	4.00	0.135
**OTSMDLFC**	13,896.63	6217.45	14,907.10	9806.62	10,639.77	5227.77	-	4.88	0.087
**PALFAMS**	14.20	4.78	15.53	3.22	15.57	3.39	-	0.83	0.659
**PALMETS**	1.23	1.30	1.20	1.13	1.37	1.45	-	0.06	0.971
**PALTEA**	11.10	15.52	7.07	7.42	6.23	5.99	-	0.55	0.758
**PRMPCD**	73.33	16.44	73.89	16.34	86.11	15.22	1.2 < 3	11.71 **	0.003
**PRMPCI**	84.72	11.80	85.83	13.16	95.28	7.79	1.2 < 3	16.54 **	<0.001
**RVPA**	0.95	0.04	0.96	0.04	0.91	0.05	1.2 > 3	18.70 **	<0.001
**SSPFSL**	6.50	0.78	6.67	1.12	7.23	1.65	1.2 < 3	5.92 *	0.050

* *p* < 0.05, ** *p* < 0.01; Source: own study.

**Table 4:** BMI vs. PASI, EASI, TAS, Mini-Cope and CANTAB by groups: Spearman’s rank correlation coefficients.

Variable	BMI Psoriasis	BMI AD	BMI Healthy Persons
**PASI**	0.121	-	-
**EASI**	-	−0.189	-
**TAS20 TOTAL**	−0.173	0.419 *	0.173
**DIF**	−0.085	0.261	0.164
**DDF**	−0.064	0.358 *	0.222
**EOT**	−0.118	0.340 *	−0.003
**Active coping**	−0.110	0.278	−0.059
**Planning**	−0.027	−0.030	−0.231
**Positive reinterpretation**	−0.029	0.132	−0.080
**Acceptance**	0.222	−0.007	−0.073
**Sense of humor**	−0.115	−0.063	0.359 *
**Turning to religion**	0.177	−0.066	−0.184
**Seeking of emotional support**	0.089	−0.086	−0.355 *
**Seeking of instrumental support**	0.114	−0.135	−0.217
**Doing something else**	0.033	−0.012	−0.135
**Denial**	−0.070	0.079	−0.195
**Venting of emotions**	−0.087	−0.127	0.006
**Use of psychoactive substances**	−0.265	0.365 *	−0.333 *
**Cessation of activities**	0.135	−0.080	0.014
**Self-blame**	−0.017	−0.107	0.260
**ERTRT**	0.214	−0.006	0.022
**ERTTH**	−0.226	0.106	0.091
**OTSMDLFC**	−0.049	−0.096	−0.034
**PALFAMS**	−0.086	−0.041	−0.032
**PALMETS**	0.262	−0.025	0.102
**PALTEA**	0.039	0.002	0.019
**PRMPCD**	0.292	0.033	0.143
**PRMPCI**	−0.156	−0.148	−0.333 *
**RVPA**	−0.103	0.185	0.063
**SSPFSL**	0.113	0.087	0.041

* *p*< 0.05; Source: own study.

## Data Availability

Data are available on reasonable request due to privacy/ethical restrictions.
